# Lipschütz Ulcer: EBV or Mycoplasma?

**DOI:** 10.1007/s13224-025-02139-z

**Published:** 2025-07-02

**Authors:** Matteo Terrinoni, Federica Adinolfi, Angelo Baldoni, Dario Rossetti, Gian Carlo Di Renzo

**Affiliations:** 1 Department of Medicine and Surgery, University of Perugia, 06129 Perugia, PG Italy; 2 Department of Obstetrics and Gynecology, “Alto Tevere” Hospital of Città di Castello, USL Umbria 1, Città di Castello, Italy; 3 Department of Obstetrics and Gynecology, Azienda Ospedaliera di Perugia, Perugia, Italy; 4 PREIS School, International and European School of Perinatal, Neonatal and Reproductive Medicine, Florence, Italy; 5 Department of Obstetrics, Gynecology and Perinatology, I.M. Sechenov First State University of Moscow, Moscow, Russia

**Keywords:** Epstein–Barr virus, Genital ulcers, Lipschütz ulcers, Mycoplasma pneumoniae, Vulvar lesions

## Abstract

**Background:**

Lipschütz’s acute vulvar ulceration is the vulvar manifestation of a systemic pathology. It is first reported in 1912 and is a non-sexually acquired condition characterized by symmetrical genital ulcers. It is an underdiagnosed condition with poor cases described in the literature. The main symptom is pain.

**Case Presentation:**

We describe the case report of a 14-year-old patient with painful genital ulcers associated with flu-like symptoms. The gynaecological examination showed the presence of multiple vulvar lesions. The diagnosis of Lipschütz ulcers was a diagnosis of exclusion.

**Conclusion:**

This rare condition must not be underestimated because of any psychophysical implication. It is mandatory to reassure the patient and her family of its benign course.

## Introduction

Lipschütz ulcer, first described in 1912, is the vulvar manifestation of a systemic pathology and it is characterized by the presence of symmetrical ulcers affecting the external genitalia, usually in sexually inactive young women < 20 years old [1].

It is the vulvar manifestation of a systemic pathology.

It is an underdiagnosed condition that usually resolves in less than 3 weeks from the onset. The main symptom is pain.

This is a case report about the clinical evolution of this rare form of vulvar ulcer in a 14-year-old patient.

## Case Presentation

A 14-year-old girl came to our attention, accompanied by her parents, complaining the appearance of painful vulvar lesions.

**Fig. 1:**
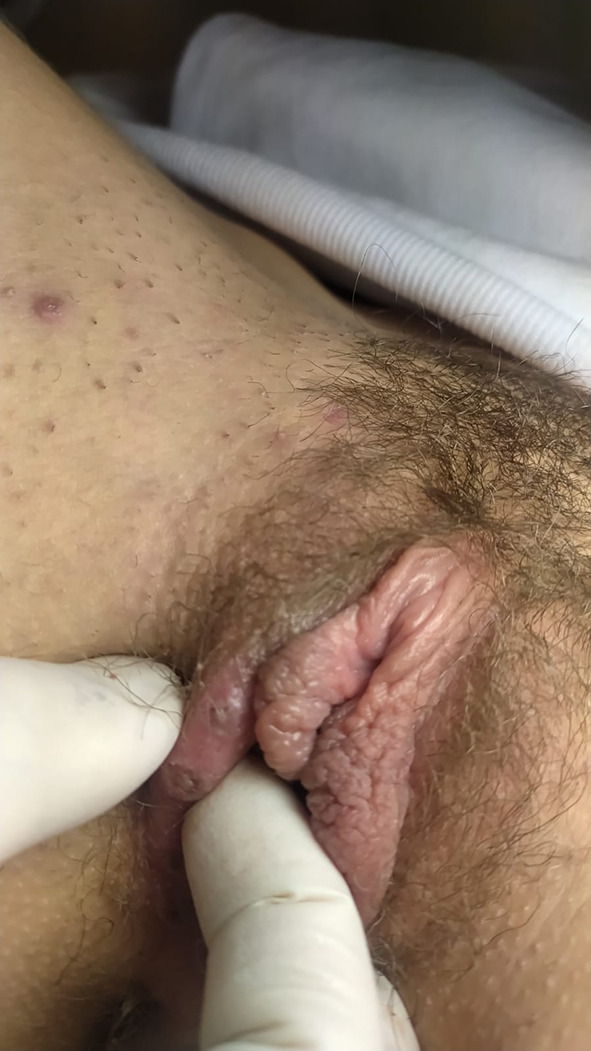
Lipschütz ulcer: right labia majora

**Fig. 2:**
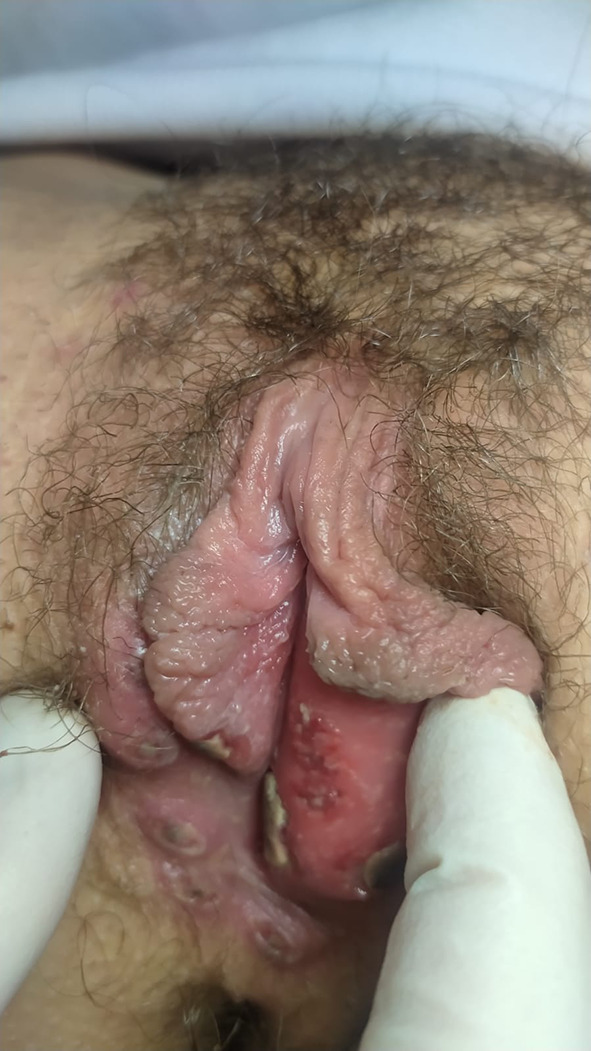
Lipschütz ulcer: left labia minora and vestibule

During the gynaecological examination, the presence of multiple ulcers localized on the labia majora and labia minora, < 1 cm in size, in different developmental states, was noted. These ulcerative lesions appeared greyish-white in colour, markedly painful, with a fibrinous background. Upon palpation, they had a soft consistency. Leucorrhoea is absent. A mild lymphopathy was appreciated in the groin. No signs of trauma is observed (Figs. 1, 2).

Absence of vesicles in the vulvar and anal area.

The patient also reported asthenia, fever, physical discomfort and headache.

Virgo patient. The absence of oral lesions in the anamnesis do not allow the diagnosis of Behçet’s disease. Concomitant modest pharyngotonsillitis and mild pharyngeal erythema. In the immediate medical history, a very recent hospitalization for Mycoplasma pneumoniae pneumonia emerged (positive throat swab, positive ELISA test). Despite this, the patient underwent blood chemistry tests, oropharyngeal swab and genital swab.

- White blood cells were normal except for mild lymphocytosis in percentage terms (66%, normal range 19–45%).
- Light increase in transaminases: Alanine aminotransferase (ALT) (56 U/I, normal range < 40 U/I), lactate dehydrogenases (LDH) 318 U/I (normal range 0–248 U/I).
- Antibodies for hepatotropic viruses: Hepatitis B surface antigen (HbsAg): negative; antibodies to Hepatitis C virus (anti-HCV test): negative; Hepatitis A immunoglobulin IgM/IgG (anti-HAV): negative.
- Haemagglutination Treponema pallidum (TPHA): negative; Venereal Disease Research Laboratory test (VDRL): negative.
- Immunoglobulin M (IgM) for Epstein–Barr Virus (EBV): positive.
- SARS-Cov-2 oropharyngeal swab: negative.
- SARS-Cov-2 quantitative reverse transcription polymerase chain reaction (RT-qPCR): negative.
- Immunoglobulin M (IgM), Immunoglobulin G (IgG) for Herpes Simplex Virus 1(HSV-1) and Herpes Simplex Virus 2 (HSV- 2): negative.
- Urethral and vaginal swabs for Bacteria, Candida, Trichomonas, Chlamydia trachomatis, Neisseria gonorrhoeae research: negative.
- Swabs performed on ulcerative lesions: negative.

Considering the anamnestic data, the oropharyngeal swab, the blood chemistry tests and the objective examination, the diagnosis of EBV infectious mononucleosis was made and then the diagnosis of Lipschütz ulcer was made. The patient underwent symptomatic therapy. The longitudinal clinical control performed at 14 days showed complete healing of the lesions and confirmed the previously made diagnosis.

## Discussion

The differential diagnosis about genital ulcers is complex: sexually transmitted diseases, inflammatory reactions and autoimmune diseases. Lipschütz ulcers are a rare entity that can affect women of any age, most frequently ≤ 20 years of age.

The clinical presentation involves the association of flu-like symptoms preceding or concomitant with genital ulcerations [2]. Among the etiological factors, viral diseases are widely the main cause, most of all Epstein–Barr virus (EBV) and less frequently other viruses, such as influenza, cytomegalovirus (CMV) and paramyxovirus. Lesions associated with bacterial infections such as Mycoplasma pneumoniae and group A streptococcus are rarer. The most accredited hypothesis suggests hypersensitivity with subsequent deposition of immune complexes and thrombosis of small vessels affecting the vulvar region.

The ulcers appear necrotic with an erythematous edge and overlying grey–black exudate [3].

The diagnosis is a diagnosis of exclusion associated with a concurrent/recent influenza-like infection.

The main criteria include: (1) acute onset of one or more painful ulcerative lesions in the vulvar region; (2) exclusion of other potential causes of the ulcer, including infectious and non-infectious processes. Minor criteria include: (1) ulcers confined to the vestibule or labia minora; (2) no history of sexual intercourse or no sexual intercourse in the past 3 months; (3) flu-like symptoms; (4) systemic infection in the last two to four weeks before the appearance of genital ulcer. The diagnosis requires both major criteria and at least two minor criteria to recommend the diagnosis of Lipschütz ulcer [4].

Failure to diagnose could lead to unnecessary and expensive diagnostic tests with the spectre of a sexually transmitted disease, cancer or abuse. There is no association between Lipschütz ulcers and sexually transmitted infections.

The evolution is benign and self-limiting and requires supportive treatment with topical anesthetics or oral analgesics, and the use of systemic corticosteroids is rarely required.

The singularity of our case must be attributed to the concomitant presence of two etiological factors which does not allow us to clarify the precise cause.

## Conclusions

Lipschütz ulcers are a rare but clinically significant cause of genital ulcers. A careful medical history is essential. They should always be taken into consideration when diagnosing vulvar lesions.

It is mandatory to reassure the patient and her family of its benign course and provide appropriate supportive therapy.

## Data Availability

Data sharing not applicable to this article as no datasets were generated or analysed during the current study.
